# Reducing Human Album Solution Use in the Pediatric Intensive Care Unit

**DOI:** 10.1097/pq9.0000000000000667

**Published:** 2023-07-10

**Authors:** Yu Inata, Etsuko Nakagami-Yamaguchi, Takeshi Hatachi, Yukie Ito, Takaaki Akamatsu, Muneyuki Takeuchi

**Affiliations:** From the * Department of Medical Quality and Safety Science, Osaka Metropolitan University Graduate School of Medicine, Osaka, Japan; † Department of Intensive Care Medicine, Osaka Women’s and Children’s Hospital, Osaka, Japan.

## Abstract

Evidence for outcome improvement is limited for using 5% human albumin solution (5% albumin) in pediatric intensive care units (PICUs). However, 5% albumin was injudiciously used in our PICU. Therefore, we aimed to decrease 5% albumin use in pediatric patients (17 years old or younger) in the PICU by 50% in 12 months to improve health care efficiency.

**Methods:**

We plotted the mean 5% albumin volume used per PICU admission monthly on statistical process control charts through 3 study periods: baseline period before intervention (July 2019–June 2020), phase 1 (August 2020–April 2021), and phase 2 (May 2021–April 2022). With intervention 1, education, feedback, and an alert sign on 5% albumin stocks began in July 2020. This intervention continued until May 2021, when we executed intervention 2, removing 5% albumin from the PICU inventory. We also examined the lengths of invasive mechanical ventilation and PICU stay as balancing measures across the 3 periods.

**Results:**

Mean 5% albumin consumption per PICU admission decreased significantly from 48.1 to 22.4 mL after intervention 1 and 8.3 mL after intervention 2, with the intervention effects persisting for 12 months. Costs associated with 5% albumin per PICU admission significantly decreased by 82%. In terms of patient characteristics and balancing measures, the 3 periods were not different.

**Conclusions:**

Stepwise quality improvement interventions, including the system change with the elimination of the 5% albumin inventory from the PICU, were effective in reducing 5% albumin use in the PICU with sustained reduction.

## INTRODUCTION

### Available Knowledge

Excessive fluid administration has been associated with poor outcomes in children.^1^ Human albumin solution (albumin) has been widely used as an alternative to crystalloids based on the belief that colloids are more likely to remain in the intravascular space and more effective in improving hemodynamics and fluid balance than crystalloids. However, based on clinical data and the revised Starling equation, the colloids used for fluid resuscitation may have little advantage over the administration of crystalloids.^2,3^ Indeed, several studies have provided evidence against the use of albumin compared with crystalloids in pediatric patients, especially those with septic shock or undergoing cardiac surgery.^4,5^ Strong evidence for utilizing albumin to improve outcomes is limited to several clinical situations,^6^ which are rare in the pediatric intensive care unit (PICU). Despite a lack of evidence of its beneficial effects, critically ill children frequently receive colloids.^7,8^

### Problem Description

Automated dispensing cabinets are rarely implemented in Japanese hospitals. Instead, commonly used drugs and fluids were stored in cabinets and made readily available in our PICU. The pharmacy restocked the cabinet daily, including 5% albumin. ICU physicians frequently used 5% albumin as an alternative to crystalloids, either as a bolus or a continuous infusion. When considering the prospect of a fluid bolus in a patient with hemodynamic instability, some of our physicians occasionally draw 5% albumin into a syringe; such behavior is not uncommon in Japan, illustrating Japan’s lag in task shifting—the rational redistribution of tasks among health workforce teams.^9^ These practices appear to have their roots in the belief that albumin is more effective than crystalloids in improving hemodynamics and/or leading to better fluid balance. Another problem is that once a continuous albumin infusion has begun, as in the case of crystalloids, unless a physician deliberately placed a discontinuation order, the nurse would renew a bottle or syringe containing the albumin without notifying a physician. Cost-conscious physicians would often be frustrated to discover that a nurse renewed an albumin bottle before realizing that they should discontinue the albumin infusion.

### Rationale

**Fig. 1.**
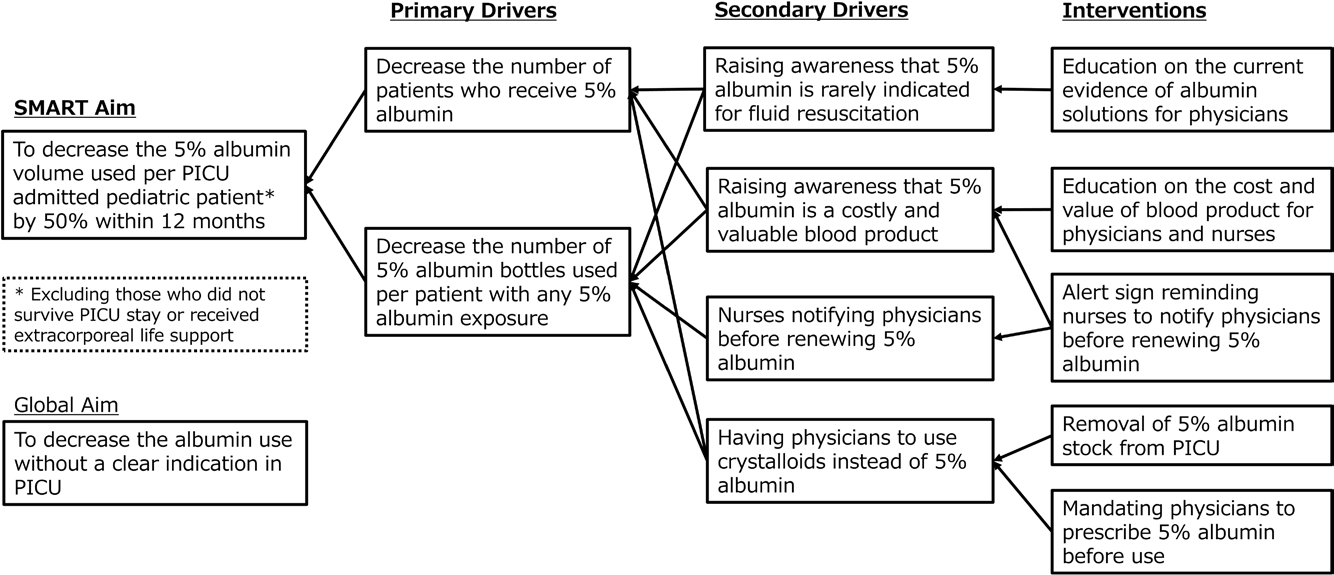
Key driver diagram.

We gathered information from our key stakeholders, the ICU physicians, and nurses. As a result, we identified problems including (1) lack of knowledge that, based on the current evidence, 5% albumin is rarely indicated for fluid resuscitation, (2) lack of awareness and/or knowledge that albumin is a costly and valuable blood product, (3) lack of system where nurses notify physicians before renewing albumin, and (4) the convenience of 5% albumin administration with no prescription required. Therefore, our quality improvement (QI) team designed a key driver diagram (Fig. 1) and implemented interventions toward these barriers.

Education, alerts, and reminders have been used to raise awareness and change health care provider behavior.^9^ However, we felt that forcing physicians to prescribe 5% albumin by removing the stock from PICU was the most effective. In a single-center QI investigation, hydroxyethyl starch, another colloid type, was significantly decreased by altering the order set.^10^ Hydroxyethyl starch was removed from the order template. As a result, it was no longer available as a default option alongside other fluid bolus options on the order template. In contrast, physicians could order it via manual search on the electronic system.

### Specific Aims

We aimed to decrease the 5% albumin volume used per patient by 50% in 12 months among pediatric patients who were 17 years or younger and survived a PICU stay without extracorporeal membrane oxygenation, hemodialysis, and plasma exchange or replacement (**Fig.1**). The QI purpose was to improve health care efficiency without worsening the patient’s clinical outcome.

### Ethical Considerations

The hospital’s ethics committee approved this study and waived the need for informed consent (approval number 1553).

## METHODS

### Context

The Osaka Women’s and Children’s Hospital is a tertiary care freestanding children’s hospital with 198 general pediatric wards. The 12-bed PICU and an 8-bed step-down unit admit approximately 800 medical and surgical patients, including nearly 200 patients undergoing cardiothoracic surgery annually. Seven to 8 attending physicians oversee the PICU and the step-down unit, along with several fellows and pediatric residents.

The PICU, like many ICUs in Japan, uses 2 distinct electronic medical records (EMRs) made by different companies: an EMR used across the hospital (the general EMR: HOPE EGMAIN-GX, Fujitsu, Tokyo, Japan) and an EMR used only in the ICU and OR (the ICU EMR: GAIA, Nihon Kohden, Tokyo, Japan). Due to the weak interoperability of the 2 EMRs, a drug administration order in the ICU EMR does not automatically generate the corresponding prescription order in the general EMR. Therefore, frequently used IV drugs and fluids, including albumin, were kept in PICU cabinets to be administered promptly without being prescribed in the general EMR. In our hospital, the PICU was the only ward stocked with albumin in the drug cabinet, but all code carts, including one in the PICU, contained 5% albumin.

### Interventions

**Fig. 2.**
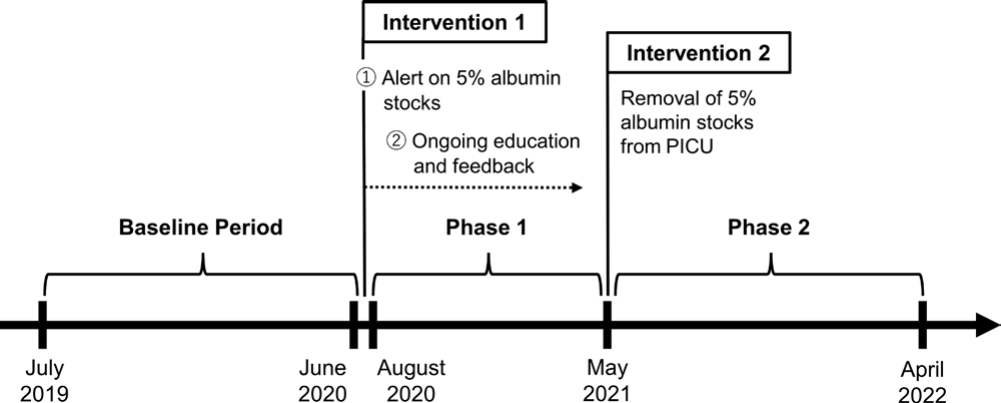
Timeline showing the baseline, phase 1, and phase 2 periods, and the intervention timing.

An extemporary team of a few staff physicians and nurse leaders led a series of interventions (Fig. 2). Although we hypothesized that complete removal of albumin from the PICU would be most effective in reducing albumin use, a drastic change of the long-standing practice would have encountered strong opposition from those who firmly believed in the clinical effectiveness of 5% albumin. The first interventions thus consisted of placing an alert sign on the albumin stock and providing staff education and feedback. The focus was more on reducing unintended and unnecessary albumin renewal than reducing albumin initiation.

### Intervention 1

A card with an alert message was placed on albumin bottles inside the cabinet so that nurses would easily notice the alert when picking a bottle. The message stated, “Human albumin solution is a blood product. Before renewal, please ask a physician if it needs to be continued (**Supplemental Digital Content 1,** An alert message card on albumin bottles, http://links.lww.com/PQ9/A496).” We also gave the staff ongoing education and feedback. We also reviewed articles reporting the potential albumin complication and the lack of evidence supporting the use of albumin for fluid resuscitation in the journal club to educate physicians. During the morning rounds, the physicians leading the QI discussed the injudicious use of albumin when it was noted. Likewise, when they observed unintended albumin renewals, they would educate the nurse on the value of albumin and encourage him/her to consult a physician before renewal.

### Intervention 2

The second short intervention consisted of 5% albumin stock elimination from the PICU and system changes that mandate a prescription for 5% albumin before use.

### Study of the Intervention(s)

To measure the intervention effectiveness, we plotted the mean 5% albumin volume used per patient admitted to the PICU on statistical process control (SPC) charts through 3 study periods: baseline (July 2019–June 2020), phase 1 (August 2020–April 2021), and phase 2 (May 2021–April 2022) (Fig. 2).

The EMR was used to review albumin use in all patients aged 17 or younger admitted to the PICU during the study period. Patients who died in the PICU or required therapies that often necessitate albumin replacement—extracorporeal membrane oxygenation, hemodialysis, and plasma exchange or replacement—were excluded from the analysis because they were potential outliers and could obscure the intervention effect in the general PICU population.

The 5% albumin use among the 3 study periods was compared in addition to the SPC chart analysis. Additionally, the percentage of patients receiving multiple 5% albumin bottles and the number of 5% albumin orders among patients with any orders were compared and examined to delineate the effect of the intervention in discouraging unnecessary albumin renewal. We also contrasted patient characteristics to assess the impact of changes in those factors on albumin use. Finally, the duration of invasive mechanical ventilation and the PICU length of stay were evaluated as balancing measures.

### Measures

The primary outcome measure was the 5% albumin volume per PICU-admitted patient. Whenever a 5 g/100 mL (5%) albumin bottle was opened for a certain patient, we calculated 100 mL as the used volume regardless of the exact volume the patient received because the primary concern was health care efficiency rather than the albumin effect on the individual patient. Similarly, 250 mL was measured after opening a 12.5 g/250 mL (5%) bottle.

Secondary outcome measures were the patient percentage exposed to any 5% albumin amount, the patient percentage exposed to multiple 5% albumin bottles, the mean number of 5% albumin bottles used among patients with 5% albumin exposure, and the total 5% albumin cost per month and per PICU admission. The cost was calculated based on the official prices approved by the Ministry of Health, Labour and Welfare in Japan; albumin costs ¥2,507 for a 5 g/100 mL (5%) bottle and ¥3,906 for a 12.5 g/250 mL (5%) bottle, whereas a commonly used 500 mL bag of crystalloid costs around ¥200 in Japan.

Balancing measures were the duration of invasive mechanical ventilation and the PICU length of stay.

### Analysis

SPC charts for trend analysis were created using Microsoft Excel and QI Macros. Established statistical control chart QI rules were applied to discerning special versus common cause variation.^11^ Basic statistics were performed by calculating the mean and median of the processes analyzed. The χ^2^ and Kruskal-Wallis tests were used to compare categorical and continuous data.

## RESULTS

**Fig. 3.**
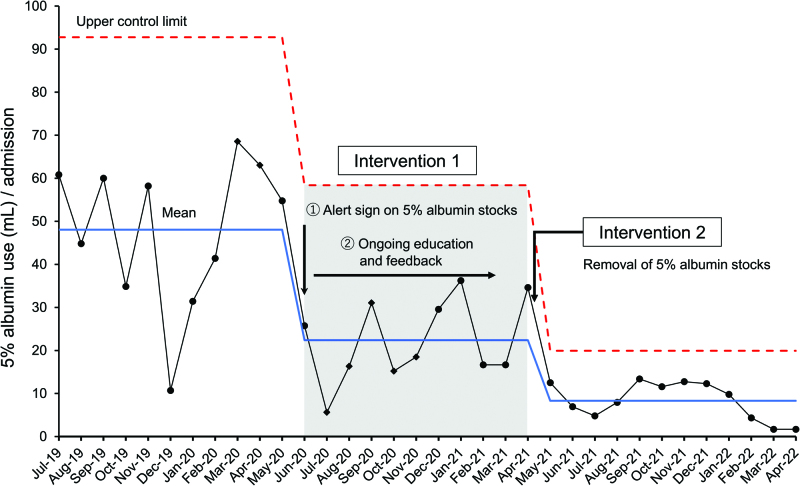
Monthly data on the use (mL) of 5% human albumin solution per PICU admission, showing trends before and after interventions.

Intervention 1 began in July 2020 and continued until May 2021, when intervention 2 was executed. As part of intervention 1, we placed the alert sign on the albumin stock in July 2020 to encourage prudent albumin use and gave nurses and physicians ongoing education and feedback. These interventions appeared effective in raising awareness of the problem among physicians and nurses. As a result, the mean 5% albumin volume per PICU admission decreased by 53%, from 48.1 to 22.4 mL, after intervention 1 (Fig. 3).

Fortuitously, several months after initiating intervention 1, hospital leadership decided to introduce a system where the transfusion department stores and dispenses albumin instead of the pharmacy to gain financial incentives from the government. Taking advantage of this opportunity, we removed the entire albumin stocks, including one in the code cart, on the rollout day of the hospital-wide albumin management system change in May 2021 (intervention 2). Physicians were now mandated to order albumin from the transfusion department before administering it to patients.

After intervention 2, the mean 5% albumin volume per PICU admission dropped by 63%, from 22.4 to 8.3 mL, for a total of 83% reduction from baseline. Furthermore, the reduction in 5% albumin use persisted for 12 months after intervention 2 (Fig. 3).

Table 1 displays 5% albumin usage and associated expenditures during the 3 study periods. The patient percentage exposed to multiple 5% albumin bottles and the mean number of 5% albumin bottles used per patient with any 5% albumin exposure decreased over time, indicating reduced 5% albumin renewal. On the other hand, the patient percentage exposed to any 5% albumin also decreased over time. As a result, the mean 5% albumin costs per PICU-admitted patient significantly decreased by 82% from ¥1,091 at baseline to ¥197 at the phase 2 period.

**Table 1.** 5% Human Albumin Solution Use and Its Cost: Comparison among 3 Periods

5% albumin use and cost	Baseline, 2019.7–2020.6	Phase 1, 2020.8–2021.4	Phase 2, 2021.5–2022.4	*P*
Patients exposed to any 5% albumin, %	15.3	11.5	4.4	<0.01
Patients exposed to multiple bottles of 5% albumin, %	10.1	5.3	1.4	<0.01
Mean number of 5% albumin bottles used per patient with any 5% albumin exposure	2.6	2.1	1.6	<0.01
Mean albumin cost per month, yen	62,927	36,497	10,776	<0.01
Mean 5% albumin costs per PICU admission, yen	1,091	642	197	<0.01

Patient characteristics and balancing measures were not significantly different among the 3 study periods: baseline (July 2019–June 2020), phase 1 (August 2020–April 2021), and phase 2 (May 2021–April 2022) (Table 2).

**Table 2.** Patient Characteristics and Clinical Outcomes: Comparison among 3 Periods

Patient characteristics and outcomes	Baseline, 2019.7–2020.6	Phase 1, 2020.8–2021.4	Phase 2, 2021.5–2022.4	*P*
Age, months	19 [4-63]	17 [4-60]	18 [4-57]	0.86
Male, N (%)	351 (51)	270 (53)	359 (61)	0.34
PIM2, %	1.1 [0.6–2.1]	1.1 [0.5–1.9]	1.1 [0.7–1.9]	0.61
PIM3, %	0.9 [0.4–1.7]	0.8 [0.3–1.5]	0.9 [0.5–1.5]	0.081
Patients undergoing cardiac surgery, N (%)	104 (15.0)	102 (19.9)	115 (17.5)	0.083
Patients receiving invasive mechanical ventilation, N (%)	419 (60.5)	317 (61.9)	411 (62.7)	0.72
Duration of invasive mechanical ventilation, days	2 [1–4]	1 [1–3]	1 [1–3]	0.15
Length of PICU stay, days	3 [2–5]	3 [2–6]	3 [2–6]	0.94

PIM, Pediatric Index of Mortality.

Numbers are expressed as median [interquartile range] unless otherwise specified.

## DISCUSSION

This single-center QI project involving a 2-phase intervention resulted in an 83% reduction in albumin consumption for pediatric patients in the PICU. It greatly improved health care efficiency by reducing 5% albumin costs per PICU-admitted patient by 82%. In addition, the effect of the system change involving the albumin removal from PICU in intervention 2 was definitive and persisted for at least 12 months.

Several studies demonstrated the effects of guiding physicians’ choices through default changes.^12^ Although the change in the albumin management system and the requirement to order albumin from the transfusion department did not forbid albumin use, they undoubtedly influenced the ICU physicians’ behavior. However, these changes may have encountered strong opposition from the staff if they were abrupt and unanticipated. Deimplementation—identifying and removing harmful, non-cost-effective, or ineffective practices based on tradition, and without adequate scientific support—often faces psychological barriers that inhibit behavior change and may require proactive mitigation strategies to address such barriers.^13–16^ Physicians may perceive deimplementation as a loss.^14^ In that regard, intervention 1, with education, feedback, and the alert sign, while being a slow and nondefinitive process, was a crucial step toward successfully implementing intervention 2. It likely helped lay the groundwork for physicians accepting the system change involving albumin removal from the PICU. Both 5% albumin renewal and initiation started to decline during the intervention 1 phase, and the intervention 2 system change met no significant opposition from the PICU physicians.

The ICU physicians’ perspectives have probably changed due to the current QI intervention. It is conceivable that the less they observed other physicians using albumin, the more likely it was for them to believe that not using albumin was “normal” and that they realized that 5% albumin was no more necessary than they initially believed.^14^ Following the 5% albumin stock removal from the PICU, the hospital removed every 5% albumin in all code carts based on the PICU physicians’ agreement.

A similar approach in the current QI activities can be applied to many hospitals in Japan since they still stock albumin in the ICU (personal correspondence). According to a recent study using Japanese nationwide inpatient record data, patients admitted to hospitals with a transfusion management system, where the transfusion department stores and dispenses albumin and promotes appropriate albumin use, were less likely to receive albumin than patients admitted to hospitals without such a system.^17^ However, the transfusion management system’s impact on albumin use in the ICU had not been elucidated. Our results indicate that albumin stock prohibition on the floor, rather than albumin management by the transfusion department *per se*, is the key driver in reducing albumin use.

In the current study, the decision to use 5% albumin was left at the individual physician’s discretion. However, in a recent study, a clinical pharmacist-led strategy intervening on all albumin orders noncompliant with institutional guidelines significantly reduced inappropriate albumin use and costs in hospitalized patients.^18^ Creating guidelines and evaluating albumin use appropriateness would be our next goal since our institution lacks institutional guidelines on albumin use and albumin stewardship.

There are several limitations to this study. First, our finding is unlikely to apply directly to places without an albumin ward inventory. Nevertheless, our strategies to guide health care providers’ choices through default changes and to address their psychological barriers in advance may apply to other situations.^12,14^ Second, the long intervention periods may have allowed changes in the patient population and the severity of the illness to confound the intervention effects. However, given the similarities in patient characteristics, such confounding is probably negligible. Third, we excluded patients from the analyses who required extracorporeal membrane oxygenation, hemodialysis, and plasma exchange or replacement and those who died in the PICU. The number of excluded cases was 88 during the study period, that is, 5% of the total patients. Due to the small number of excluded patients, evaluating how current QI interventions affected them was impossible.

## CONCLUSIONS

The system change involving the removal of albumin stock from the PICU reduced 5% albumin use and improved health care efficiency. A similar framework of interventions may be applied to the control of other fluids or medications. Consideration should be given to raising awareness of QI activities among the staff before implementing any drastic system change.

## ACKNOWLEDGMENT

Assistance with the study: The authors would like to thank Mr. Kenji Hirai for his assistance with the data curation.

## DISCLOSURE

The authors have no financial interest to declare in relation to the content of this article.

## Supplementary Material
