# Supplementary material for: Reducing Human Album Solution Use in the Pediatric Intensive Care Unit
Source: Pediatr Qual Saf. 2023 Jul 10;8(4):e667. doi: 10.1097/pq9.0000000000000667 (PMC10332825; doi:10.1097/pq9.0000000000000667)

Human albumin solution is a blood product.
Before renewal, please ask a physician if it needs to be continued.

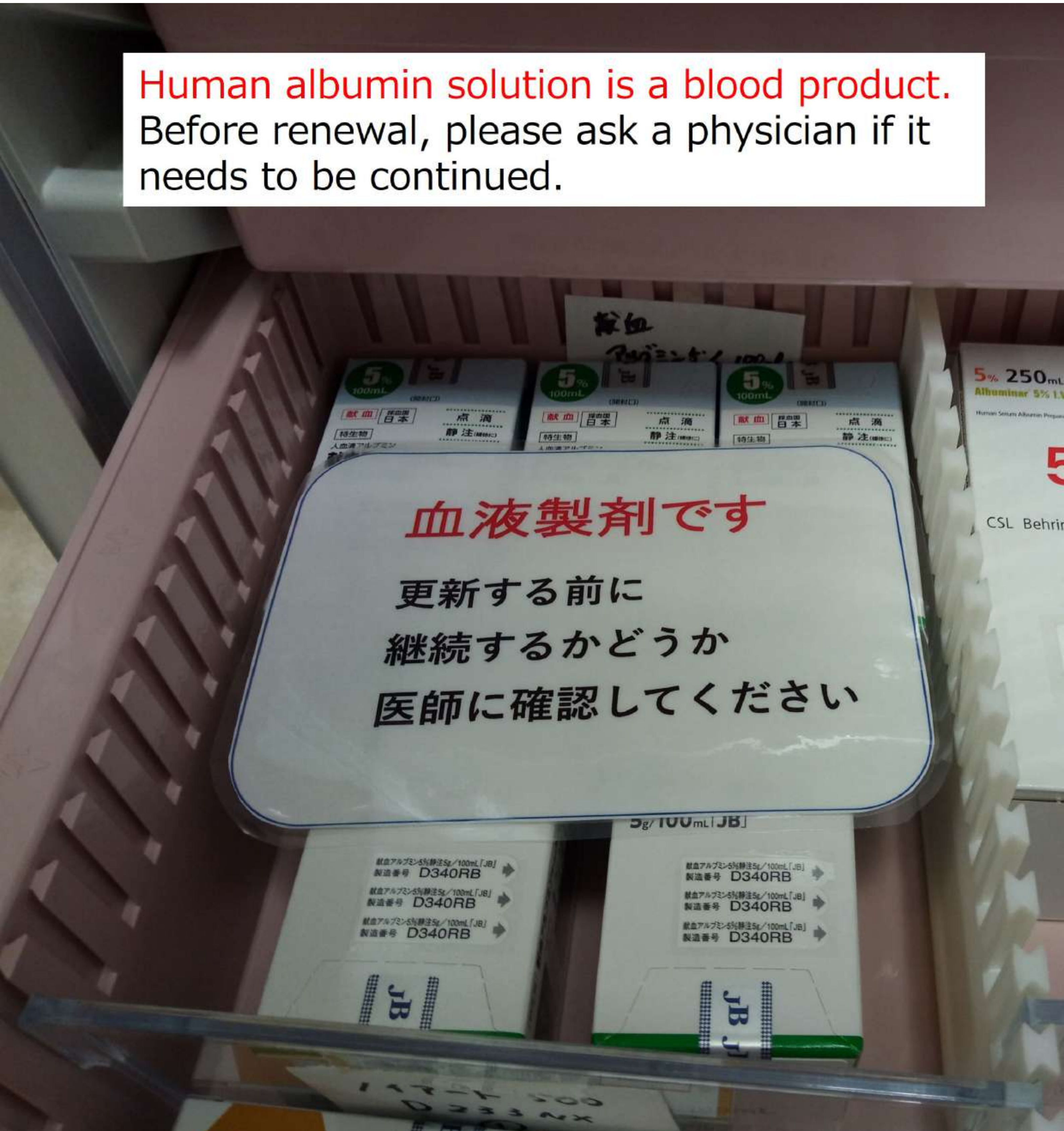

Supplement: Supplementary file 1 [file pqs-8-e667-s001.pdf]
